# Peripheral precocious puberty in a 4-month-old infant associated with an ESR1 variant: a case report

**DOI:** 10.1093/omcr/omag194

**Published:** 2026-10-17

**Authors:** Fadi Ayyash, Noor alrawajfeh, Asmaa Monther Al Quraan, Hadeel Othman Alqurieny, Abdelrazaq Ahmad Alyasin, Juana Hani AbuRahmeh, Nama' Abdullah Bany Amer, Sura Al-Rawabdeh, Waseem Al Mefleh, Sondos Harahshih

**Affiliations:** Department of Pediatrics, Queen Rania Al Abdullah Hospital for Children, King Hussein Medical City, King Abdullah II Street 226, Amman 11855, Jordan; Department of Internal Medicine, Prince Hamza Hospital, Habis Al-Majali Street, Al-Hashmi Al-Shamali, Amman, Amman Governorate 11947, Jordan; Department of Pediatrics, Queen Rania Al Abdullah Hospital for Children, King Hussein Medical City, King Abdullah II Street 226, Amman 11855, Jordan; Department of Pediatrics, Queen Rania Al Abdullah Hospital for Children, King Hussein Medical City, King Abdullah II Street 226, Amman 11855, Jordan; Department of Pediatrics, Queen Rania Al Abdullah Hospital for Children, King Hussein Medical City, King Abdullah II Street 226, Amman 11855, Jordan; Department of Internal Medicine, Prince Hamza Hospital, Habis Al-Majali Street, Al-Hashmi Al-Shamali, Amman, Amman Governorate 11947, Jordan; Department of Pediatrics, Prince Hamza Hospital, Habis Al-Majali Street, Al-Hashmi Al-Shamali, Amman 11947, Jordan; Department of Pathology, King Hussein Medical Center, Royal Medical Services, Amman, Amman Governorate 11855, Jordan; Department of Pediatrics, Queen Rania Al Abdullah Hospital for Children, King Hussein Medical City, King Abdullah II Street 226, Amman 11855, Jordan; Department of Pediatrics, Queen Rania Al Abdullah Hospital for Children, King Hussein Medical City, King Abdullah II Street 226, Amman 11855, Jordan

**Keywords:** precocious puberty, ESR1 variant, estrogen receptor, whole-exome sequencing, peripheral precocious puberty, tamoxifen

## Abstract

Precocious puberty (PP) refers to pubertal onset before 8 years in females and 9 years in males. We present a rare case of a 6-year and 7-month-old Jordanian girl with peripheral PP onset at 4 months of age. Clinical evaluation revealed vaginal spotting, premature thelarche, advanced bone age, and recurrent uterine bleeding requiring multiple transfusions. Imaging demonstrated a progressive cervicovaginal mass and endometrial polyps. Histopathology identified atypical hyperplasia/endometrioid intraepithelial neoplasia. Gonadotropins remained persistently suppressed, excluding central PP. Whole-exome sequencing (WES) identified a heterozygous likely-pathogenic ESR1 missense variant (NM_000125.3; c.1610A > C; p.Tyr537Ser), previously linked to estrogen-independent receptor activation in breast cancer. Tamoxifen produced partial clinical response with subsequent recurrence; the patient is now on combined letrozole–tamoxifen therapy. This case highlights the value of WES in unexplained PP and suggests that gain-of-function ESR1 variants may drive peripheral PP through receptor hypersensitivity.

## Introduction

Precocious puberty (PP) is defined as pubertal signs before 8 years in girls and 9 years in boys [1]. PP is classified as central (CPP), driven by early hypothalamic–pituitary-gonadal axis activation, or peripheral (PPP), resulting from autonomous gonadal or adrenal sex hormone secretion independent of gonadotropin stimulation [2]. Recognised PPP causes include McCune–Albright syndrome (MAS), primary hypothyroidism, adrenal pathology, and ovarian lesions.

PP onset in infancy particularly as early as 4-months is exceedingly rare. The ESR1 gene encodes estrogen receptor alpha (ERα), central to reproductive development. Gain-of-function ESR1 variants, well-characterized in hormone-resistant breast cancer, have not previously been described as a germline cause of PP in infancy. We report the first documented association between a germline ESR1 variant and peripheral PP in an infant.

## Case report

A 6-year and 7-month-old girl, born to first-degree consanguineous parents with no prenatal complications or family history of breast cancer, presented with vaginal spotting and premature thelarche onset at 4 months. Examination revealed pallor, Tanner stage III breast development, stage II pubic hair, no café-au-lait spots, and no clitoromegaly. Growth was markedly accelerated (Figs 1 and 2). There was no exogenous estrogen or medication exposure.

**Figure 1:**
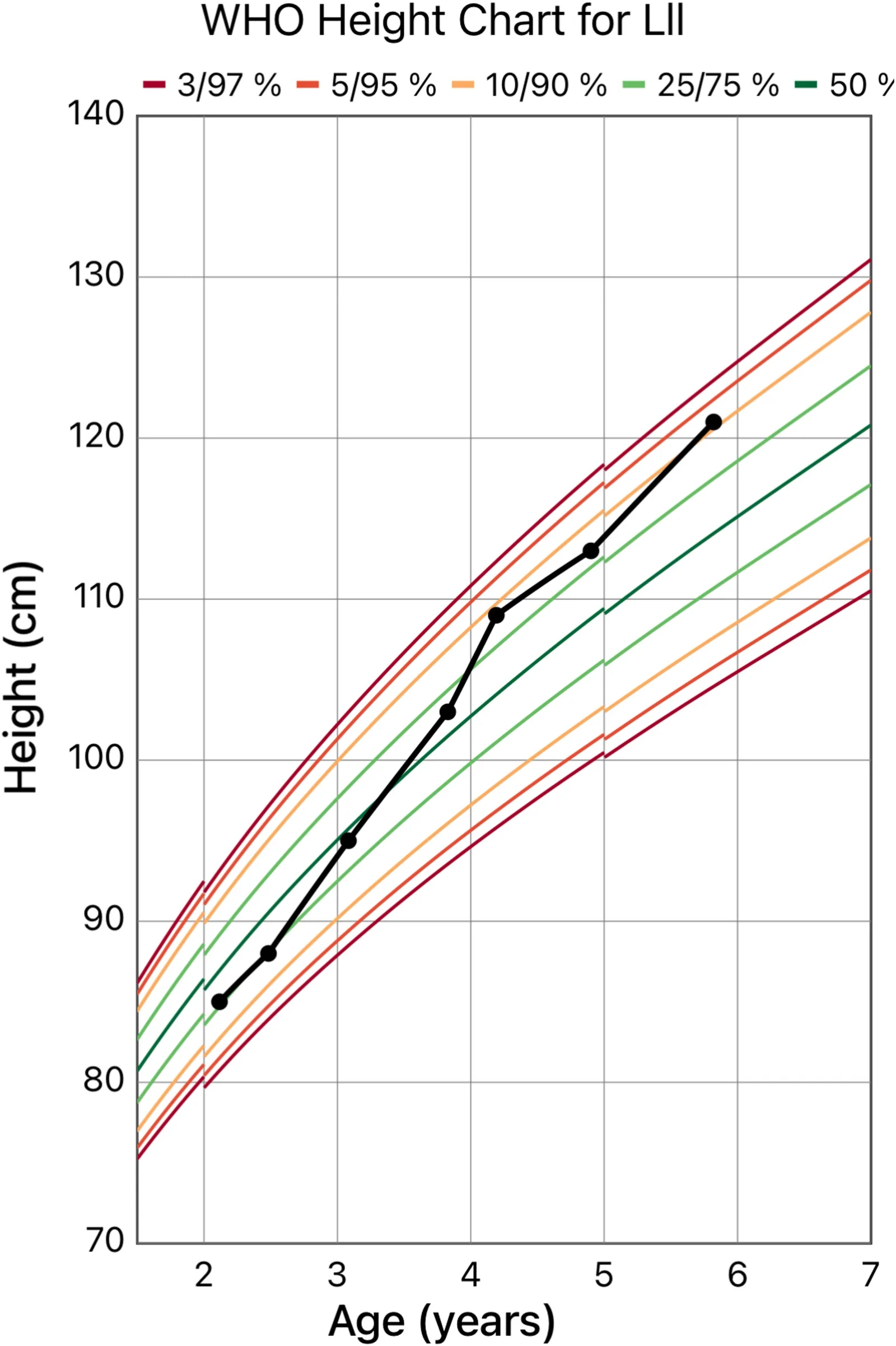
WHO height-for-age growth chart demonstrating accelerated linear growth.

**Figure 2:**
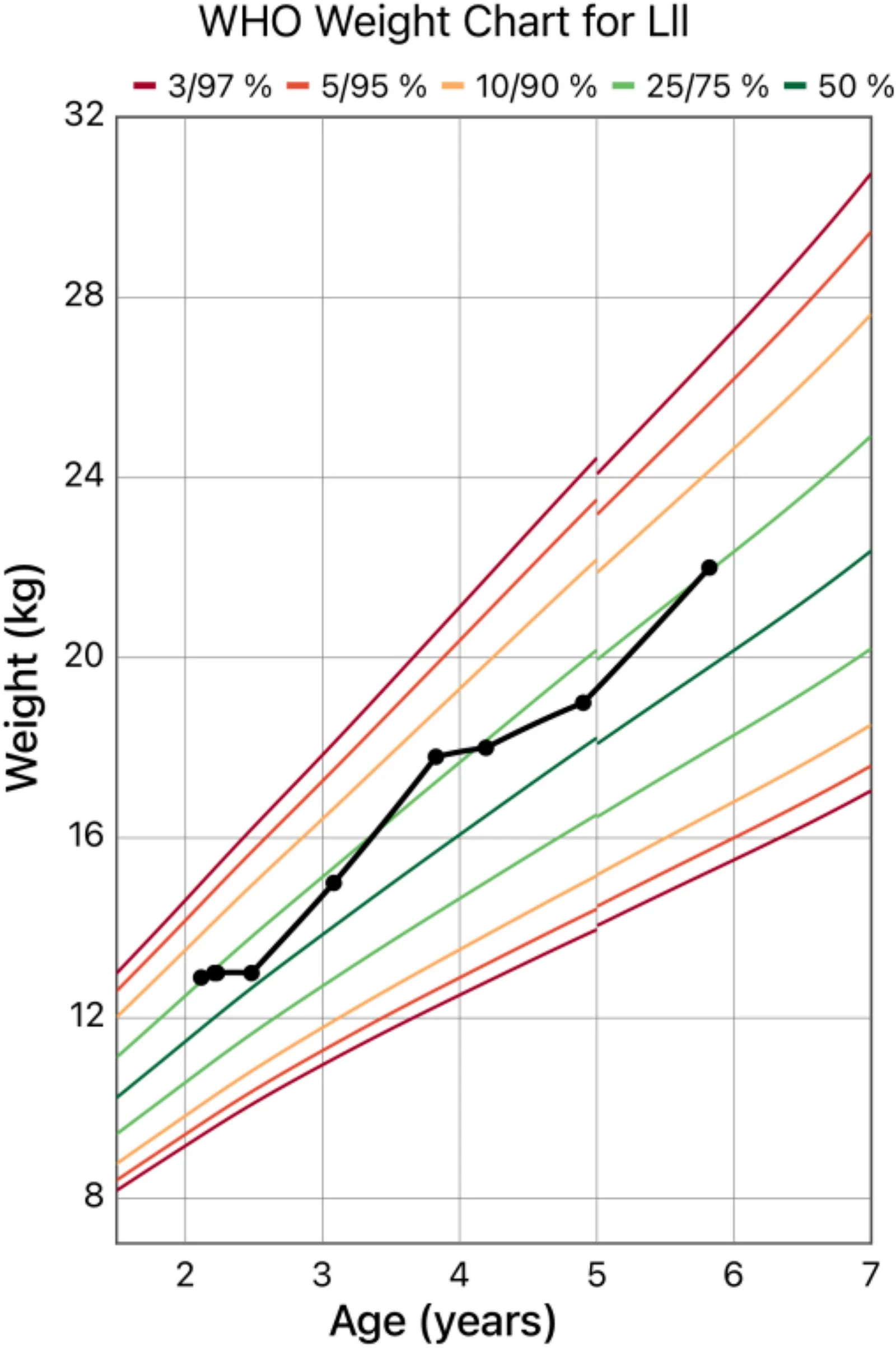
WHO weight-for-age growth chart demonstrating accelerated weight gain.

### Laboratory findings

LH remained persistently low (0.1 IU/L); FSH followed a similar pattern. A GnRH stimulation test showed minimal LH increment (peak 0.3 IU/L), LH/FSH ratio 0.25–0.27, incompatible with CPP. Estradiol was transiently elevated (peak 12.3 pmol/L). AFP declined from 98 IU/mL (2020) to normal by 2023; β-hCG was undetectable. Thyroid function, DHEAS, 17-hydroxyprogesterone, ACTH, and prolactin were normal. Recurrent severe anaemia (haemoglobin nadir 47 g/L) required five red cell transfusions between 2021 and 2023 (Table 1).

**Table 1:** Serial Hematological findings demonstrating a decline in packed cell volume (2021–2023).

**PCV (L/L)**	**Hb (g/L)**	**Date**	**Action**
0.165 (Low)	48 (Severely Low)	20/9/2021	Admitted for PRBC infusion
0.277 (Low)	84 (Low)	23/9/2021	–
0.160 (Low)	47 (Severely Low)	3/1/2022	Admitted for PRBC infusion
0.220 (Low)	65 (Very Low)	10/1/2022	Admitted for PRBC infusion
0.310 (Low-normal)	100 (Low)	6/5/2022	–
0.250 (Low)	72 (Low)	8/5/2022	–
0.172 (Low)	53 (Severely Low)	25/7/2022	Admitted for PRBC infusion
0.360 (Low-normal)	119 (Low-normal)	12/8/2022	–
0.370 (Normal)	110 (Low)	17/8/2022	–
0.170 (Low)	52 (Severely Low)	4/5/2023	Admitted for PRBC infusion

### Imaging findings

Pituitary MRI (2020, 2022) was normal (Fig. 3). Skeletal survey and whole-body CT showed no lytic lesions, organomegaly, or lymphadenopathy; bone age advanced from ~ 5-years and 5-months at age 4 to nearly 9 years by May 2025 (Fig. 4). Pelvic ultrasound was normal in 2021; a cervicovaginal mass appeared in early 2023 (42 × 33 × 25 mm), enlarging to 82 × 77 × 46 mm by May 2023. Pelvic MRI (January 2022) characterised a 3.8 × 2.7 × 4.4 cm heterogeneously enhancing vaginal mass, raising the differential of botryoid rhabdomyosarcoma and yolk sac tumour, both excluded histologically. Tamoxifen (September 2023) led to mass regression (25 × 26 × 18 mm), but re-enlargement with increased vascularity occurred by February 2025.

**Figure 3:**
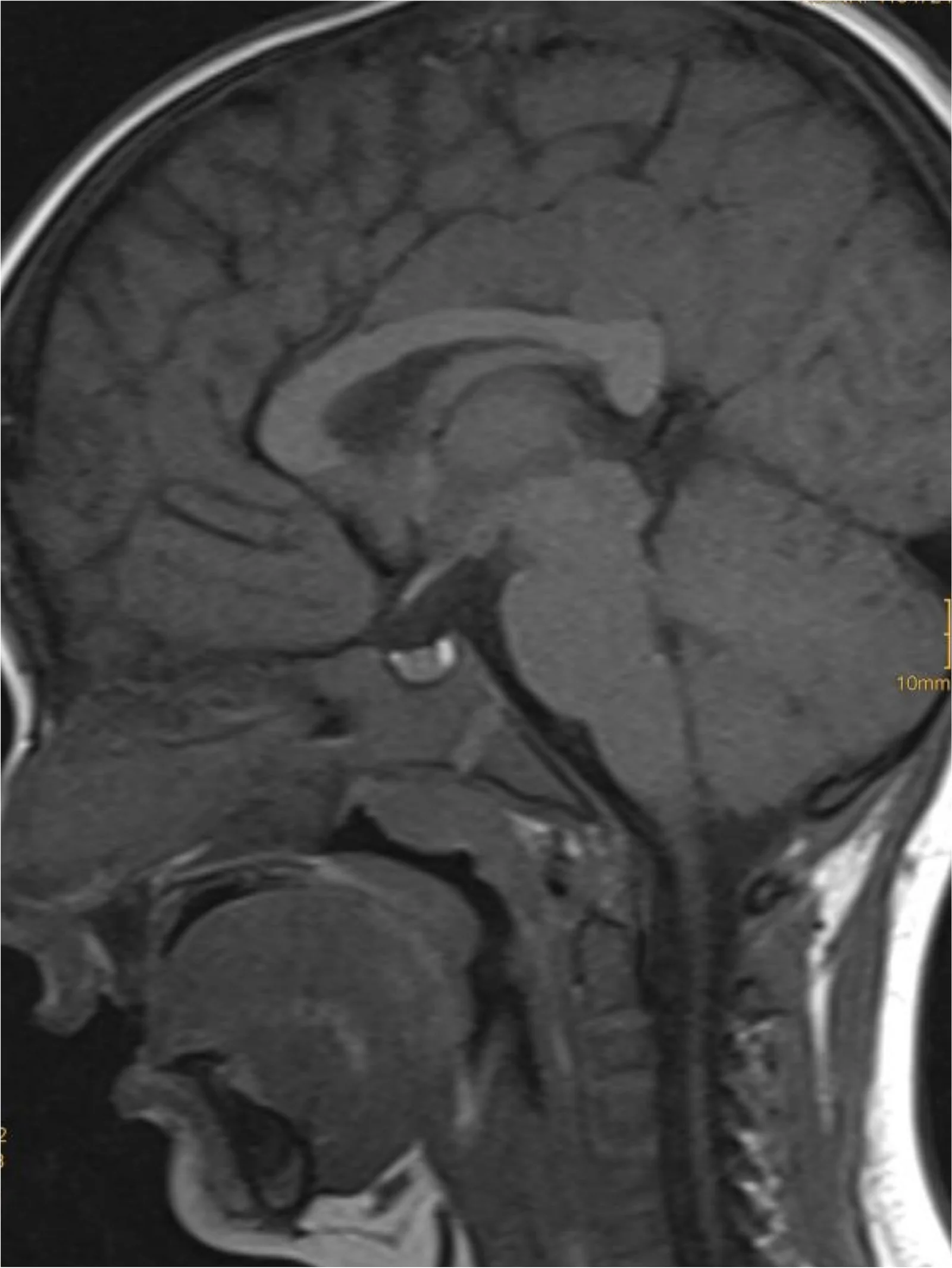
MRI of the pituitary gland (October 2022) showing no abnormalities, excluding central causes of precocious puberty.

**Figure 4:**
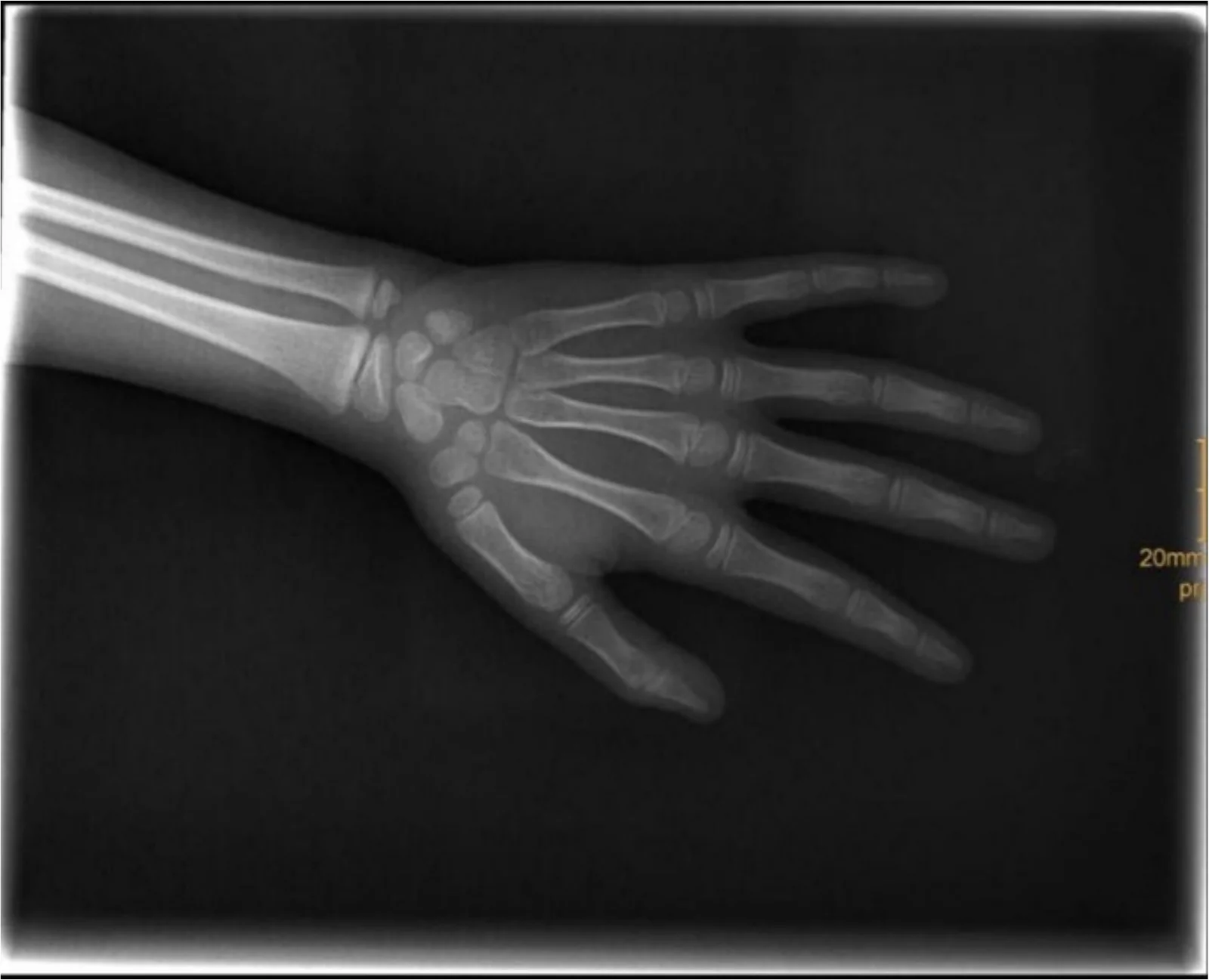
Left wrist X-rays showing progressive skeletal maturation: Bone age of approximately 5-years and 5-months at age 4 years, advancing to nearly 9 years by May 2025, with open growth plates, consistent with precocious puberty.

### Histopathology

Laparoscopy confirmed normal ovaries and a bulky uterus. Hysteroscopy removed multiple endometrial polyps (Fig. 5). Biopsies (2021–2023) showed hyperplastic polyps; atypical hyperplasia/endometrioid intraepithelial neoplasia (AH/EIN) was identified in August 2022 and May 2023 (Fig. 6). Rhabdomyosarcoma, germ cell tumour, and haematological malignancy were excluded.

**Figure 5:**
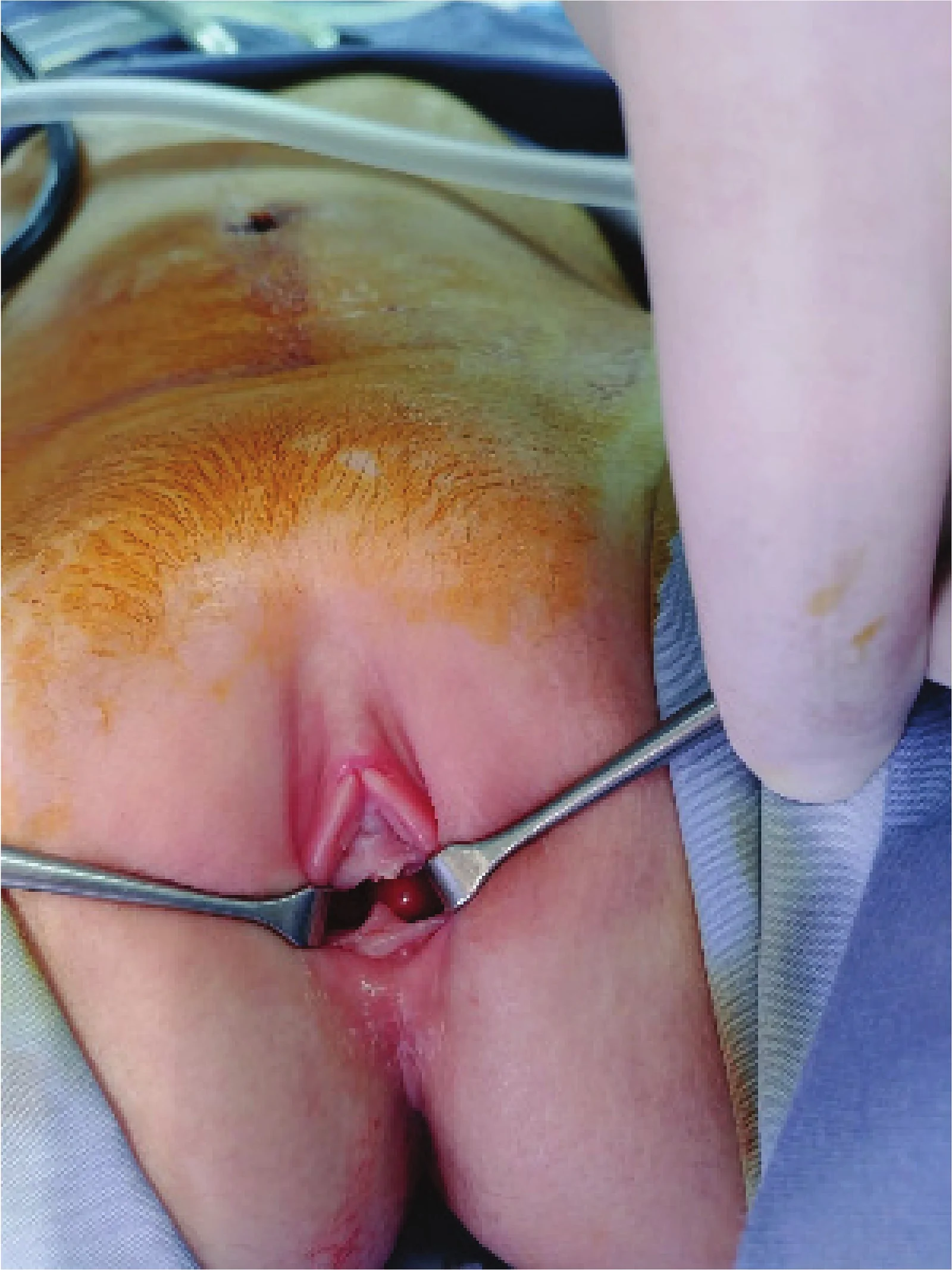
Intraoperative view (2023) showing a protruding vaginal polyp visible at the vaginal introitus, with surrounding tissue prepared for surgical excision.

**Figure 6:**
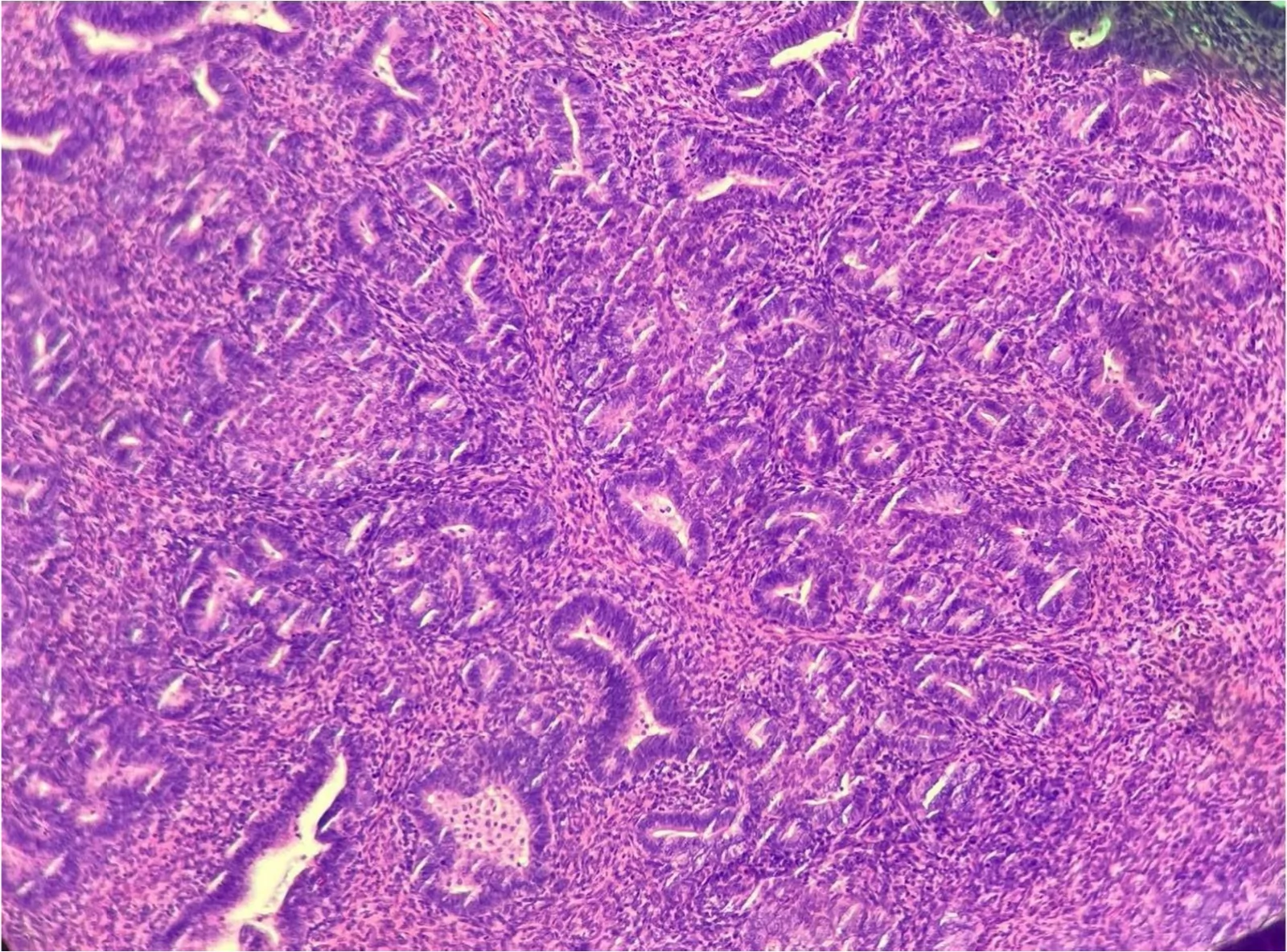
Histopathology demonstrating atypical endometrial hyperplasia/endometrioid intraepithelial neoplasia (AH/EIN).

### Genetic findings

Whole-exome sequencing identified a heterozygous likely-pathogenic ESR1 missense variant (NM_000125.3; c.1610A>C; p.Tyr537Ser; Chr6:152419923), located in a ClinVar mutational hotspot [3] (Table 2). This variant drives estrogen-independent ERα activation in breast cancer; its germline effect may confer receptor hypersensitivity in this patient.

**Table 2:** Whole-exome sequencing findings: Likely pathogenic ESR1 variant.

**Variant details**	
**Gene ID**	**ESR1**
**Transcript**	NM_000125.3
**Location**	Chr6:152419923
**HGVS c.**	c.1610A>C
**HGVS p.**	p.Tyr537Ser
**Coverage**	43:39
**Zygosity**	Heterozygous
**Inheritance Pattern**	Autosomal Dominant
**Disease Association**	Breast Cancer, Estrogen Resistance/Sensitivity
**Classification**	Likely Pathogenic
**Classification Database**	ClinVar, Varsome, and Franklin

### Management

Letrozole (age 3 years, six months) failed to control bleeding. Tamoxifen (September 2023) achieved symptom relief and mass regression but recurrence occurred by February 2025. The patient is currently on combined letrozole–tamoxifen therapy.

## Discussion

This case is notable for three reasons: PP onset at 4 months of age, a likely-pathogenic germline ESR1 variant identified by whole-exome sequencing (WES), and AH/EIN in a young child a combination not previously reported. We emphasise that the ESR1 variant is a plausible contributing factor, not a proven cause; causation requires functional validation beyond the scope of a single case report.

Persistently suppressed gonadotropins and a flat GnRH stimulation test confirmed a peripheral mechanism, excluding CPP. Autonomous ovarian cysts, the most common PPP cause in infants [4] were excluded by laparoscopy showing normal bilateral ovaries. MAS was excluded clinically: no café-au-lait lesions, no fibrous dysplasia on skeletal survey, and no organomegaly on whole-body CT [5]. Adrenal and germ cell aetiologies were excluded by normal DHEAS, 17-hydroxyprogesterone, undetectable β-hCG, and age-appropriate AFP decline.

The ESR1 p.Tyr537Ser variant confers constitutive, ligand-independent ERα activity [6]. Low-level estradiol fluctuations are consistent with intermittent follicular activity, which receptor hypersensitivity may have amplified. WES provided diagnostic resolution where PCR-based methods with limited sensitivity would not [7, 8]. Tamoxifen, chosen to target the receptor-mediated mechanism, showed partial response supporting this pathway, though prospective validation is needed.

AH/EIN in this setting is unprecedented. Estrogen receptor overexpression is linked to endometrial polyp formation [9]; sustained ERα activation likely underlies the progressive endometrial pathology observed. Endometrial surveillance should be considered in children with gain-of-function ESR1 variants.

### Strengths and limitations

Strengths include the exceptional rarity of this phenotype, rigorous multi-modal exclusion of established PPP aetiologies, WES-confirmed genetic finding, AH/EIN documentation, and five-year longitudinal follow-up. Limitations include pending parental genetic testing (de novo vs. inherited origin undetermined), lack of functional variant validation, and unknown long-term endometrial malignancy risk. Regarding GNAS testing: MAS is caused by a post-zygotic somatic mosaic GNAS mutation, and standard testing on blood or saliva has poor sensitivity due to low allelic frequency in non-affected tissues—a negative blood-based result would therefore not have excluded MAS. Definitive molecular exclusion would require testing of affected tissue such as bone lesion or café-au-lait skin biopsy; critically, neither was present in this patient, further corroborating the clinical exclusion of MAS on both phenotypic and molecular-methodological grounds. This case highlights the value of WES in unexplained infantile PPP; a germline ESR1 gain-of-function variant represents a novel, hypothesis-generating mechanism, and combined letrozole–tamoxifen therapy warrants prospective evaluation [10].
